# Skeletal Muscle-Specific Ablation of γ_cyto_-Actin Does Not Exacerbate the *mdx* Phenotype

**DOI:** 10.1371/journal.pone.0002419

**Published:** 2008-06-11

**Authors:** Kurt W. Prins, Dawn A. Lowe, James M. Ervasti

**Affiliations:** 1 Department of Biochemistry, Molecular Biology, and Biophysics, University of Minnesota, Minneapolis, Minnesota, United States of America; 2 Department of Physical Medicine and Rehabilitation, University of Minnesota, Minneapolis, Minnesota, United States of America; Hospital Vall d'Hebron, Spain

## Abstract

We previously documented a ten-fold increase in γ_cyto_-actin expression in dystrophin-deficient skeletal muscle and hypothesized that increased γ_cyto_-actin expression may participate in an adaptive cytoskeletal remodeling response. To explore whether increased γ_cyto_-actin fortifies the cortical cytoskeleton in dystrophic skeletal muscle, we generated double knockout mice lacking both dystrophin and γ_cyto_-actin specifically in skeletal muscle (ms-DKO). Surprisingly, dystrophin-deficient *mdx* and ms-DKO mice presented with comparable levels of myofiber necrosis, membrane instability, and deficits in muscle function. The lack of an exacerbated phenotype in ms-DKO mice suggests γ_cyto_-actin and dystrophin function in a common pathway. Finally, because both *mdx* and ms-DKO skeletal muscle showed similar levels of utrophin expression and presented with identical dystrophies, we conclude utrophin can partially compensate for the loss of dystrophin independent of a γ_cyto_-actin-utrophin interaction.

## Introduction

Duchenne muscular dystrophy (DMD) is a progressive muscle wasting disease affecting approximately 1 in every 3500 males [1]. Afflicted males experience a severe dystrophy marked by wheelchair dependence in the early teens and death due to cardiac and respiratory failure in the mid to late twenties [2]. DMD results from the loss of dystrophin [3], a 427 kDa protein localized to the sub-sarcolemmal space of muscle cells [4]. Dystrophin functions to stabilize muscle cell membranes by binding costameric γ_cyto_-actin [5] and the transmembrane dystroglycan complex [6]–[8], thereby linking the costameric cytoskeleton to the extracellular matrix (ECM) [9], [10]. Dystrophin-deficiency leads to muscle cell necrosis/regeneration and muscle weakness [11] due to a heightened susceptibility to muscle contraction-induced damage [12].

Although the dystrophin-deficient *mdx* mouse [13] provides a genetic homologue for DMD, the dystrophy of the *mdx* mouse is less severe than presented by DMD patients. Identification of compensatory proteins responsible for attenuating the phenotype in *mdx* mice may be useful for developing new therapeutic targets for DMD. For example, utrophin, the autosomal homologue of dystrophin, is upregulated in *mdx* mice [14]–[18] and is believed to mitigate the dystrophin-deficient phenotype due to functional overlap between utrophin and dystrophin [7], [19]. Accordingly, mice lacking both utrophin and dystrophin (*mdx*/*utrn* ^−/−^) exhibit a more severe Duchenne-like dystrophy marked by cardiomyopathy and premature death [20], [21]. Moreover, transgenic overexpression of utrophin rescues all known phenotypes of the *mdx* mouse [22]. Collectively, these results suggest increased utrophin expression can partially compensate for the loss of dystrophin in the *mdx* mouse.

While utrophin can functionally replace dystrophin, there is evidence suggesting alternative pathways between the ECM and cytoskeleton are fortified in dystrophin-deficient muscle. Levels of α7 integrin, a transmembrane protein that complexes with adapter proteins to link the actin cytoskeleton to the ECM, are increased in both DMD patients and the *mdx* mouse [23]. The severe phenotype of mice lacking both dystrophin and α7 integrin [24] and the ability of transgenic α7 integrin overexpression to increase lifespan in *mdx*/*utrn* ^−/−^ mice [25] suggests increased α7 integrin expression fortifies a parallel structural link between the ECM and the cytoskeleton in dystrophin-deficient muscle. In addition, plectin, a large cytoskeletal linker protein, was recently reported to link the dystrophin-glycoprotein complex to the intermediate filament cytoskeleton and plectin levels are also reported to be elevated in dystrophin-deficient muscle [26]. In summary, these data suggest loss of dystrophin results in a cytoskeletal remodeling response to bolster the weakened attachment of the ECM to the cytoskeleton.

We recently demonstrated γ_cyto_-actin expression is elevated approximately ten-fold in dystrophin-deficient muscle and hypothesized that the upregulated γ_cyto_-actin functions to reinforce the weakened cytoskeleton by interacting with costameric proteins such as utrophin, the α7 integrin complex, and plectin [27]. Although the function of γ_cyto_-actin in dystrophic muscle has yet to be determined, we showed γ_cyto_-actin expression is required for muscle cell viability as muscle-specific γ_cyto_-actin knockout mice develop a progressive myopathy associated with myofiber necrosis and regeneration along with costamere disorganization and functional deficits [28].

To determine if elevated γ_cyto_-actin expression stabilizes parallel linkages between the ECM and cytoskeleton in dystrophin-deficient skeletal muscle, we generated mice lacking both γ_cyto_-actin and dystrophin by breeding the conditional *Actg1* allele [28] to the *mdx* background (ms-DKO). No significant differences were measured in dystrophic histological parameters, membrane permeability, and muscle performance when *mdx* and ms-DKO mice were compared, suggesting γ_cyto_-actin and dystrophin function in a common pathway. Increased plectin expression was not found to explain the lack of an exacerbated phenotype in ms-DKO mice. However, utrophin expression was equivalently elevated in *mdx* and ms-DKO skeletal muscle and co-purified with β-dystroglycan. These results indicate utrophin can partially abrogate dystrophic phenotypes in *mdx* skeletal muscle in the absence of a direct link to γ_cyto_-actin filaments.

## Results and Discussion

### Expression and localization of cytoplasmic actins in mdx and ms-DKO skeletal muscle

**Figure 1:**
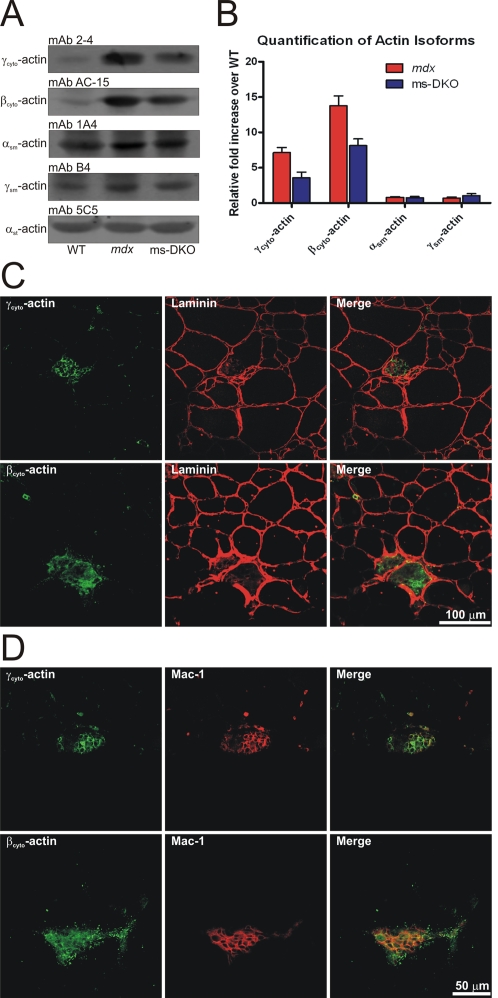
Expression and localization of actin isoforms in skeletal muscle. (A) Representative western blots of actin isoforms from actin-rich elutes from wt, *mdx*, and ms-DKO skeletal muscle. (B) Quantification of differential actin isoform expression normalized to α_st_-actin. Error bars represent SEM. (C) Ten micron thick cryosections from ms-DKO quadriceps muscle stained with antibodies to β_cyto_-actin, γ_cyto_-actin, and laminin to show focal regions of strong cytoplasmic actin immunoreactivity. (D) Ten micron cryosections from ms-DKO quadriceps show colocalization between a marcophage marker (Mac-1) and both cytoplasmic actins.

To assess the effects of increased γ_cyto_-actin expression in dystrophin-deficient muscle, mice harboring the floxed *Actg1* allele [28] and an HSA-Cre transgene [29] were bred to the *mdx* background to generate mice lacking γ_cyto_-actin and dystrophin in skeletal muscle (ms-DKO). Subsequently, expression levels of actin isoforms were determined by western blot analysis of actin extractions from skeletal muscle (Fig. 1A). Consistent with previous findings [27], one-year old *mdx* mice showed increased γ_cyto_-actin expression in skeletal muscle extracts compared to wt (7.1±0.7 fold increase). However, one-year old ms-DKO mice surprisingly showed elevated γ_cyto_-actin expression in skeletal muscle extracts compared to wt (3.6±0.8 fold increase, Fig. 1B). Both *mdx* and ms-DKO mice showed dramatic elevations in β_cyto_-actin expression in skeletal muscle extracts when compared to wt (13.8±1.4 and 8.1±0.9 fold respectively) (Fig. 1 B). However, no changes in expression levels of either α_sm_-or γ_sm_-actin were measured (Fig. 1 A and B).

To determine what cell type was responsible for increased γ_cyto_-actin and β_cyto_-actin expression in *mdx* and ms-DKO skeletal muscle extracts, immunofluorescence analysis of quadriceps cross sections was conducted. Small pockets of strong immunoreactivity were observed for both cytoplasmic actins, which appeared to be macrophages invading necrotic fibers (Fig. S1 and Fig. 1 C). Colocalization between a macrophage marker (Mac-1) and both cytoplasmic actins was observed (Fig. S1 and Fig. 1 D), suggesting the elevated cytoplasmic actin expression detected in *mdx* and ms-DKO skeletal muscle extracts was in part due to macrophage infiltration.

**Figure 2:**
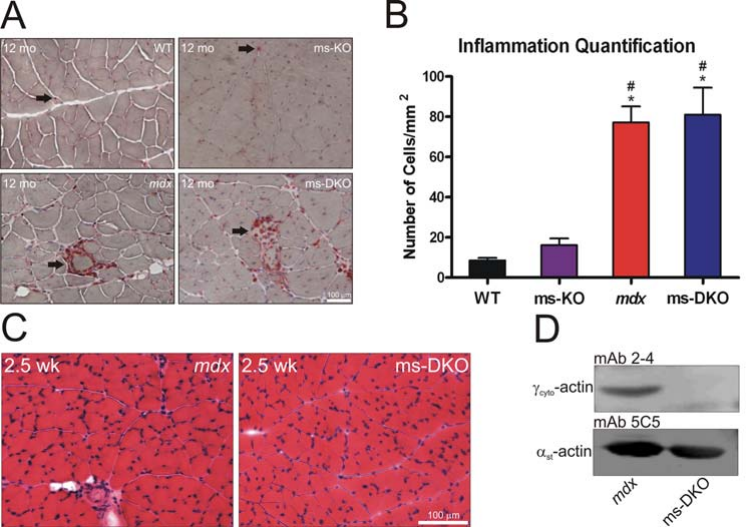
γ_cyto_-actin expression correlates with inflammation. (A) Acid phosphatase stained skeletal muscle sections from wt, γ_cyto_-actin muscle-specifc knockout (*Actg1* ms-KO), *mdx*, and ms-DKO. Arrows indicate acid phosphatase positive macrophage. (B) Quantification of macrophage infiltrants in wt, *Actg1* ms-KO, *mdx*, and ms-DKO muscle sections. The asterisk denotes a statistically significant difference from wt, pound sign denotes statistically significant difference from *Actg1* ms-KO (p<0.05). Error bars represent SEM. (C) H&E-stained quadriceps sections from 2.5 week old *mdx* and ms-DKO. Both mice show minimal histological abnormalities. (D) Western blots of actin-elutes from 2.5 week old *mdx* and ms-DKO skeletal muscle. γ_cyto_-actin is not detected in ms-DKO muscle prior to the onset of dystrophy.

We next quantified the number of macrophages present in wild type, γ_cyto_-actin muscle-specific knockout [28] (*Actg1* ms-KO), *mdx*, and ms-DKO skeletal muscle to determine if γ_cyto_-actin expression correlates with inflammatory cell infiltration. Because wt skeletal muscle shows low levels of γ_cyto_-actin expression and *Actg1* ms-KO skeletal muscle shows no γ_cyto_-actin expression, we expected to observe low levels of acid-phosphatase positive macrophages in wt and *Actg1* ms-KO skeletal muscle. As expected, the number of macrophages present in wt and *Actg1* ms-KO skeletal muscle was significantly less than the number of macrophages present in *mdx* and ms-DKO skeletal muscle (Fig. 2 A and B). Because *mdx* and ms-DKO skeletal muscle showed identical levels of macrophage infiltration, we conclude that approximately half of the γ_cyto_-actin expressed in *mdx* skeletal muscle extracts (*mdx* 7.1 fold increase vs. ms-DKO 3.6 fold increase over wt) can be attributed to macrophage infiltration.

To more definitely test the hypothesis that residual γ_cyto_-actin expression in ms-DKO skeletal muscle was solely due to macrophage infiltration, we measured γ_cyto_-actin expression in muscle extracts from 2.5 week old mice, which is prior to the onset of dystrophy (Fig. 2C). Western blot analysis of actin extractions of skeletal muscle from 2.5 week mice showed detectable γ_cyto_-actin expression in *mdx* skeletal muscle and not ms-DKO skeletal muscle (Fig. 2D). These results demonstrate that γ_cyto_-actin expression detected in ms-DKO skeletal muscle extracts was due to macrophage infiltration.

### The mdx phenotype is not exacerbated by muscle-specific ablation of γ_cyto_-actin

**Figure 3:**
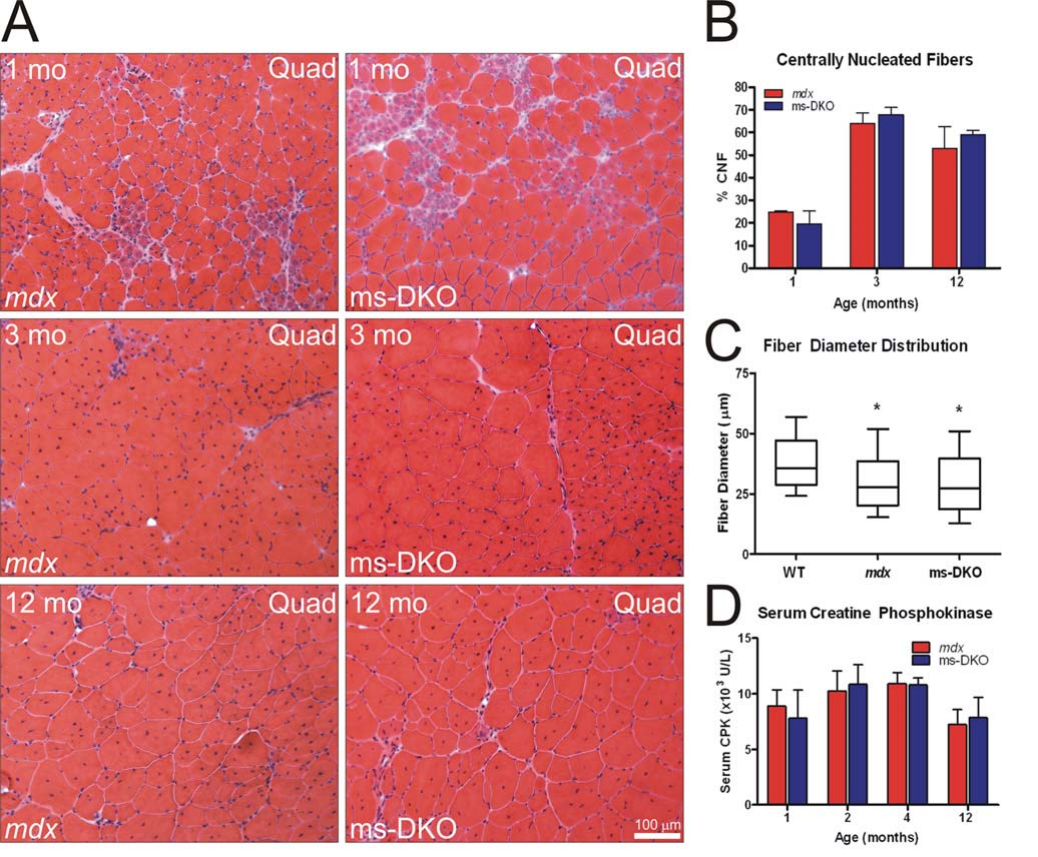
Dystrophic parameters were not exaberated in ms-DKO mice. (A) H&E-stained quadriceps sections from *mdx* and ms-DKO at 1, 3, and 12 months of age. Both mice show comparable levels of myofiber necrosis. (B) Quantification of proportion of centrally nucleated fibers in quadricepts muscle of *mdx* and ms-DKO mice at 1, 3, and 12 months of age. (C) Box and whisker blots showing the distribution of myofiber diameters from 12 month old triceps muscle from wt, *mdx* and ms-DKO mice. An asterisk denotes a significant difference in mean fiber diameter from wt mice (p<0.05). (D) Analysis of membrane permeability by quantifying serum levels of creatine phosphokinase. Error bars represent SEM in (B) and (D).

To determine if ms-DKO mice were more dystrophic than *mdx* mice, muscle cell death and regeneration was quantified by determining the proportion of centrally nucleated fibers in quadriceps muscles at 1, 3, and 12 months of age. At all timepoints examined, *mdx* and ms-DKO mice showed comparable levels of histopathology (Fig. 3A) and equivalent levels of muscle cell death and regeneration (Fig. 3B). Accordingly, the mean muscle-fiber diameter of *mdx* (31.13±0.499 µm) and ms-DKO (29.99±0.479 µm) triceps muscles was significantly smaller than the mean muscle-fiber diameter in wt triceps muscle (38.37±0.464 µm) (Fig. 3C). Muscle cell membrane fragility of *mdx* and ms-DKO did not differ as demonstrated by comparable levels of serum phosphocreatine kinase (Fig. 3D).

**Figure 4 pone-0002419-g004:**
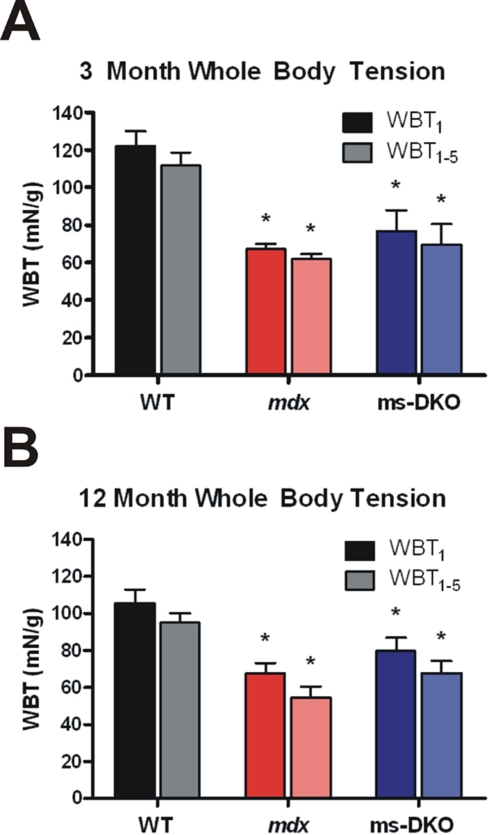
Analysis of force production *in vivo*. Whole body tension analysis on 3 month old (A) and 11–12 month old mice (B). Averages of the maximal pulling force (WBT_1_) and the top five pulling forces (WBT_1–5_) are represented graphically. Error bar represents the SEM. Asterisk indicates a statisically significant different outcome from wt (p<0.05).

To determine how loss of both γ_cyto_-actin and dystrophin affected muscle performance *in vivo*, whole body tension analysis was conducted on mice at three months and twelve months of age. At three months of age, wt mice (WBT_1_ 122.2±7.7 mN/g and WBT_1–5_ 111.8±6.9 mN/g) produced significantly more pulling force than both *mdx* (WBT_1_ 67.2±2.7 mN/g and WBT_1–5_ 61.8±2.7 mN/g) and ms-DKO mice (WBT_1_ 76.8±11.1 mN/g and WBT_1–5_ 69.6±10.8 mN/g). At twelve months of age, wt mice (WBT_1_ 105.5±7.2 mN/g and WBT_1–5_ 94.7±5.3 mN/g) generated significantly more pulling force than both *mdx* (WBT_1_ 67.6±5.4 mN/g and WBT_1–5_ 54.3±6.1 mN/g) and ms-DKO mice (WBT_1_ 79.5±7.3 mN/g and WBT_1–5_ 67.7±6.4 mN/g). The force generated by *mdx* mice was not significantly different from the force generated by ms-DKO mice at either age examined (Fig. 4).

**Figure 5:**
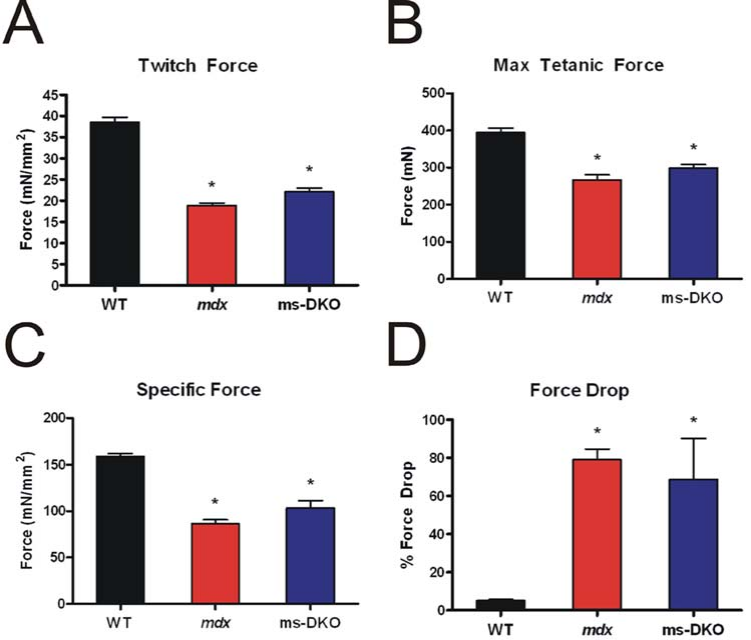
Contractile properties of isolated extensor digitorum longus muscles. Examination of normalized twitch force (A), maximal tetanic force (B), normalized maximal tetanic force (C), and susceptibility to damage caused by eccentric contractions (D). Both *mdx* and ms-DKO extensor digitorum longus muscles showed significant decrements when compared to wt extensor digitorum longus muscles. (*) indicates p<0.05 when compared to wt.

To more precisely probe muscle function, contractile properties of isolated extensor digitiorum longus muscles were determined. Consistent with the results from *in vivo* force analysis, *mdx* and ms-DKO muscle showed similar deficiencies in twitch force (Fig. 5A), maximal force production (Fig. 5B), and normalized maximal force production (Fig. 5C) when compared to wt. In addition, susceptibility to damage caused by eccentric contractions (Fig. 5D) was comparable in *mdx* and ms-DKO muscle. Taken together, the finding that *mdx* and ms-DKO muscle function is indistinguishable both *in vivo* and *ex vivo* indicates increased γ_cyto_-actin expression does not improve *mdx* muscle function. Collectively, the results of Figs. 3–5 demonstrate that the established parameters of dystrophin-deficiency in *mdx* mice were not significantly worsened by skeletal muscle-specific ablation of γ_cyto_-actin.

### Assessment of compensatory proteins in mdx and ms-DKO skeletal muscle

**Figure 6:**
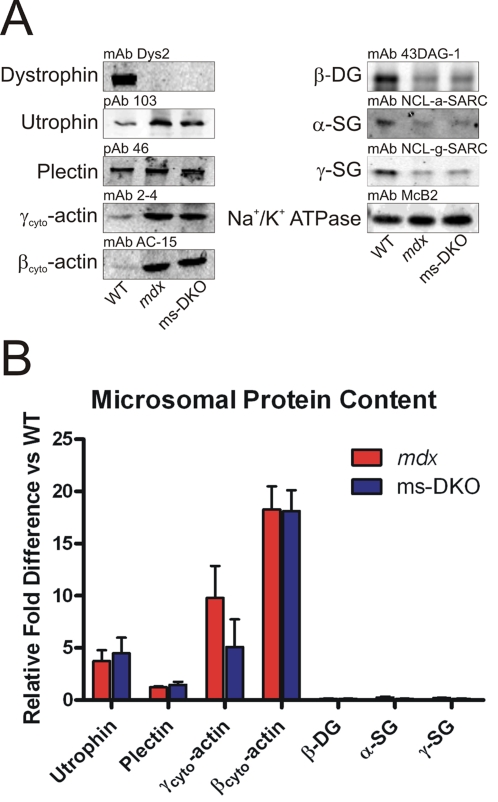
Assessment of dystrophin-associated protein content in KCl-washed microsomal preparations. (A) Representative western blots of proteins present in KCl-washed microsomal preparations. (B) Quantification of differential protein expression, Na^+^/K^+^ ATPase was used to normalize protein load. Error bars represent SEM.

To assess expression levels of dystrophin-associated glycoproteins (DAG), KCl-washed microsomes were prepared from wt, *mdx*, and ms-DKO mice for western blot analysis. α-Sarcoglycan, γ-sarcoglycan, and β-dystroglycan expression levels were similarly reduced approximately 75–80% in *mdx* and ms-DKO microsomes when compared to wt microsomes (Fig. 6). The finding that utrophin and not plectin expression levels were increased in *mdx* and ms-DKO microsomes when compared to wt microsomes (3.7±1.0 and 4.5±1.5 fold increases respectively), suggested that increased utrophin expression stabilizes low levels of DAG at *mdx* and ms-DKO membranes (Fig. 6). In agreement with a previous report [27], we observed elevated γ_cyto_-actin levels in *mdx* microsomes when compared to wt microsomes (9.8±3.0 fold increase). However, γ_cyto_-actin was also elevated in ms-DKO microsomes when compared to wt microsomes (5.1±2.6 fold increase), which suggested nonmuscle cell types contributed to approximately half of the observed increase in γ_cyto_-actin expression in *mdx* microsomes. Contaminants by nonmuscle cells types also likely explains the elevated levels of β_cyto_-actin in *mdx* and ms-DKO microsomes when compared to wt microsomes (18.3±2.2 and 18.1±2.0 fold increases respectively) (Fig. 6).

**Figure 7:**
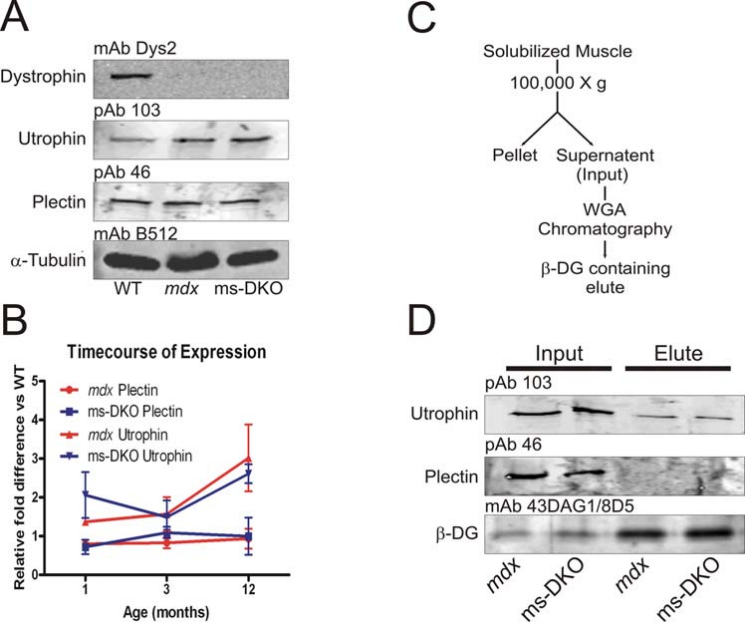
Comparison of utrophin and plectin in dystrophic muscle. (A) Representative western blots from SDS-extracted skeletal muscle from wt, *mdx*, and ms-DKO mice. (B) Quantification of utrophin and plectin expression in skeletal muscle of *mdx* and ms-DKO mice. Fold difference is determined by comparing *mdx* and ms-DKO to wt. Error bars represent standard error of the mean. (C) Schematic representation of method used to determine what proteins co-purify with β-dystroglycan. (D) Western blot analysis of the input fraction and wheat germ agglutinin elution fraction of digitonin solubilized *mdx* and ms-DKO skeletal muscle. Utrophin, and not plectin, co-purified with β-dystroglycan.

To further investigate whether plectin or utrophin may compensate for the loss of dystrophin, relative expression levels in wt, *mdx*, and ms-DKO skeletal muscle was examined by western blot analysis of SDS-extracted skeletal muscle. At all ages examined, only utrophin expression was elevated in *mdx* and ms-DKO skeletal muscle when compared to wt skeletal muscle (Fig. 7 A and B). Next, we subjected solubilized *mdx* and ms-DKO skeletal muscle extracts to wheat-germ agglutinin chromatography to determine if plectin or utrophin co-purified with β-dystroglycan (Fig. 7C). Only utrophin co-purified with β-dystroglycan in *mdx* skeletal muscle and ms-DKO skeletal muscle (Fig. 7D). In summary, these data suggest utrophin compensates for the loss of dystrophin through a γ_cyto_-actin independent mechanisms.

### Concluding remarks

Identifying proteins that can functionally compensate for the loss of dystrophin is important because strategies to upregulate such proteins may have therapeutic potential for the treatment of DMD. Recently, we identified a ten-fold increase in γ_cyto_-actin expression in dystrophin-deficient muscle and hypothesized that elevated γ_cyto_-actin expression may partially strengthen dystrophin-deficient membranes by fortifying parallel links between the ECM and cytoskeleton [27]. To determine if increased γ_cyto_-actin expression stabilizes the cortical actin cytoskeleton in dystrophin-deficient skeletal muscle, we generated mice lacking dystrophin and conditionally lacking γ_cyto_-actin in skeletal muscle (ms-DKO). Surprisingly, the phenotype of *mdx* and ms-DKO mice was identical as demonstrated by comparable levels of muscle cell death and regeneration, muscle membrane fragility, and muscle weakness. These results suggest increased γ_cyto_-actin expression does not fortify dystrophin-deficient muscle cell membranes, but γ_cyto_-actin and dystrophin lie in the same functional pathway. Consistent with previous results, utrophin co-purified with β-dystroglycan [15] and showed increased expression in *mdx* skeletal muscle [14]–[18]. We also showed utrophin co-purified with β-dystroglycan and showed expression levels comparable to *mdx* in ms-DKO skeletal muscle. Collectively, these results imply utrophin does not need to interact with γ_cyto_-actin to attenuate dystrophic phenotypes in *mdx* skeletal muscle.

## Materials and Methods

### Generation of mice

Mice harboring the conditional *Actg1* allele were described previously [28] and subsequently backcrossed a minimum of five generations to the C57BL/6 background. These mice were crossed to mice expressing cre recombinase under control of the human α_sk_-actin promoter (HSA-Cre mice were provided by Dr. Judith Melki, INSERM, France) [29] to generate mice that were homozygous for the floxed *Actg1* allele and hemizygous for HSA-Cre. These mice were backcrossed to the *mdx* background two generations to isolate mice with the genotype *Actg1* ^flox/flox^ HSA-Cre *mdx* or *Actg1* ^flox+neo/flox+neo^ HSA-Cre *mdx*. Both mice showed similar phenotypes so their results were pooled. Genotypes of the *Actg1* locus and presence of the cre transgene were determined using PCR as described [28]. All animals were housed and treated in accordance with the standards set forth by the University of Minnesota Institutional Animal Care and Use Committee.

### Antibodies

Antibodies to γ_cyto_-actin were described earlier (pAb 7577 and mAb 2-4) [27]. A polyclonal antibody (pAb) to β_cyto_-actin was generated by injecting rabbits with a peptide mimicking the amino terminus of β_cyto_-actin (2963). pAb 2963 was then affinity purified using platlet actin (Cytoskeleton Inc catalogue number APHL99) as described previously [30]. Monoclonal antibodies to β_cyto_-actin (AC-15 catalogue number A1978), α_sm_-actin (1A4 catalogue number A5228), α_st_-actin (5C5 catalogue number A2172), α-tubulin (B512 catalogue number T6074), and laminin (4H8-2 catalogue number L0663) were purchased from Sigma. The monoclonal γ_sm_-actin antibody (B4) was provided as a kind gift from Dr. James Lessard. The monoclonal Mac-1 antibody (CD 11b) was provided as a kind gift from Dr. Melissa Spencer. The polyclonal utrophin antibody (103) was provided from Dr. Stanley Froehner. The polyclonal plectin antibody (46) was a kind gift from Dr. Gerhard Wiche. The dystrophin (catalogue number NCL-DYS2), α-sarcoglycan (catalogue number NCL-a-SARC), and the γ-sarcoglycan (catalogue number NCL-g-SARC) were purchased from Novacastra. The β-dystroglycan antibody (mAb 43DAG-1, catalogue number VP-B205) was purchased from Vector Laboratories. The monoclonal Na^+^/K^+^ ATPase antibody (McB2) was described previously [31]. Infrared dye-conjugated anti-mouse and anti-rabbit antibodies were purchased from LI-COR Biosciences (catalogue numbers 926-32223 and 926-32210). Alexa-488- or 568-conjugated anti-rabbit and anti-rat 2° antibodies were purchased from Molecular Probes (catalogue numbers A11034 and A11077).

### Muscle Extracts

Muscle was harvested from anesthetized mice immediately following cervical dislocation and snap frozen in liquid N2. SDS-extracts of muscle were performed as described [27]. Protein concentration was determined using the BioRad DC Protein Assay (catalogue numbers 500-0111, 500-0112, and 500-0116). KCl-washed microsomal preparations were generated as described [32] and protein concentration was quantified using a Lowry Assay (Pierce catalogue number 23240). Actin preparations from skeletal muscle were collected by subjecting muscle to a low-salt extraction and DNase-1 amplification protocol [27].

### Western blot analysis and quantification

To determine changes in protein expression in total muscle and microsomal fractions, 25 µg of protein was subjected to SDS-PAGE and transferred to nitrocellulose. Nitrocellulose membranes were washed/blocked in a 5% milk solution in phosphate buffered-saline (PBS) for one hour at room temperature. Membranes were then incubated with primary antibodies overnight at room temperature (primary antibody dilutions: mAb 2-4 (1∶1000), mAb AC-15 (1∶500), mAb 1A4 (1∶250), mAb B4 (1∶250), mAb 5C5 (1∶2000), mAb DYS2 (1∶50), pAb 103 (1∶250), pAb 46 (1∶3000), mAb 43DAG-1 (1∶50), mAb NCL-a-SARC (1∶50), mAb NCL-g-SARC (1∶50), mAb McB2 (1∶100), and mAb B512 (1∶1000)). Membranes were washed in 5% milk solution in PBS ten minutes two times at room temperature and then incubated with IR-dye conjugated secondary antibodies (1∶10,000 dilution) for 30 minutes at room temperature. Then, membranes were washed in a 0.1% triton solution in PBS for ten minutes two times to remove excess secondary antibody. Western blots were imaged and quantified with an Odyssey Infrared Imaging System (LI-COR Biosciences catalogue number 9201-01). The normalizing signal in SDS-extracts was α-tubulin while Na^+^/K^+^ ATPase was used in microsomal fractions.

### Immunofluorescence microscopy

Individual muscles were dissected, coated in OCT (TissueTek catalogue number 4583), and then frozen in melting isopentane. Ten micron transverse sections were cut on a Leica CM3050 cryostat, air dried, then fixed in 4% paraformaldehyde for 10 minutes. Sections were washed with PBS, blocked in 5% goat serum for 30 min, and incubated with 1° antibodies overnight at 4° C (primary antibody dilutions: pAb 7577 (1∶50), pAb 2963 (1∶1) mAb 4H8-2 (1∶250), and mAb Mac-1 (1∶100)). Sections were then washed/blocked with 5% goat serum for 10 min 3 times and then incubated with Alexa-488- or 568-conjugated 2° antibodies (1∶1000 dilution) for 30 min at 37°C. Then, sections were washed with PBS and coverslips were applied with a drop of Anti-Fade Reagent (Molecular Probes catalogue number P36930). Confocal images were collected on a Bio-Rad MRC 1000 scan head mounted on an upright Nikon Optishot microscope at the University of Minnesota Biomedical Image Processing Lab. Images were equivalently processed using Adobe Photoshop.

### Assessment of dystrophic parameters

Ten micron thick cryosections of quadriceps, tibialis anterior, and triceps were stained with hematoxylin and eosin-phloxine as described [33]. Four images from different areas of the muscle section were collected on a Zeiss Axiovert 25 microscope using ImagePro Software. These images were imported into Scion Image to determine the proportion of centrally nucleated fibers (800–1000 fibers counted/muscle) at 1, 3, and 12 month old mice (n = 4 for each genotype at each timepoint). Fiber diameter distribution was determined in 12 month old triceps sections as described [33] from at least 700 fibers/genotype. Membrane permeability was determined by quantifying serum creatine kinase levels on Vitros CK DT slides (Ortho-Clinical Diagnostics catalogue number DT1975580) using a Kodak Ektachem DT60 analyzer.

### Quantification of inflammation

Ten micron thick cryosections from either quadriceps or gastrocnemius (4 sections per genotype) were subjected to an Acid Phosphatase stain. Briefly, sections were incubated in Acid Phosphatase buffer (98 mM Napthol AS-BI Phosphate, 0.046 M Sodium Acetate, 0.15% Pararosaniline, and 0.58 M Sodium Nitrite pH 5.0) for two hours at 37°C. Sections were washed extensively and then counterstained with Gills Hematoxylin and mounted in Permount (Fisher Scientific catalogue number SP15-100). Montages of muscle sections were collected on a Zeiss Microscope mounted with a Leica DFC300 FX camera. The entire muscle section was examined and the number of Acid Phosphatase-positive macrophage were counted and normalized to section area (mm^2^) using Image Pro Plus Software.

### Whole body tension

Mice were subjected to whole body tension as described [28]. At 3 months of age 5 wild type, 5 *mdx*, and 4 ms-DKO mice were subjected to analysis. At 11 to 12 months of age 5 wild type mice, 7 *mdx*, and 7 ms-DKO mice were analyzed.

### Contractile properties of isolated extensor digitorum longus

11–12 month old mice were anesthetized and the extensor digitorum longus muscle was removed (n = 4 mice per genotype). The proximal tendon was attached to 4-0 suture silk to a dual-mode muscle lever system (model 300B-LR Aurora Scientific, Aurora, ON, Canada). Muscles were equilibrated for 10 minutes in a bath assembly containing Krebs-Ringer-bicarbonate buffer (119 mM NaCl, 5.0 KCl mM, 1.0 MgSO_4_ mM, 12.25 NaHCO_3_ mM, 1.0 CaCl_2_ mM, 1.0 mM KH_2_PO_4_, 10.0 mM glucose, 0.17 mM leucine, 0.10 mM isoleucine, 0.20 mM valine plus 10 µg/ml gentammicin sulfate and 0.10 U/ml insulin) at 25°C while being constantly oxygenated with 95% O_2_/5% CO_2_ gas. Then, the resting tension was set to 0.4 g and twitch force was determined by stimulating the muscle with a 0.5 ms pulse at 150 V (Grass stimulator, Grass Telefactor, Warwick, RI). Thirty seconds later the muscle was stimulated to twitch again and the greater of two contractions was recorded. Tetanic contractions were elicited by stimulating the muscle with 150 V for 200 ms at 180 Hz. The greater of two contractions separated by two minutes of recovery time was recorded. To determine damage caused by eccentric contractions, muscles were subjected to five lengthening contractions at three minute intervals. Each ECC consisted of a maximal tetanic stimulation for 200 ms accompanied by a stretch of 0.5 L_o_/s to give a total stretch of 0.1L_o_. Force drop was calculated as ((ECC1-ECC5)/ECC1)). Results from both EDL muscles of one mouse were averaged to give a single data point.

### Wheat germ agglutinin chromatography

To determine interactions with β-dystroglycan, 100 mg of skeletal muscle was solubulized in 1 mL of 1% digitonin solution (1% Digitonin, 50 mM Tris HCl, 500 mM NaCl, and protease inhibitor [Roche catalogue number 4693116]). These samples were then centrifuged at 100,000*g* for 40 minutes and the soluble fraction was added to agarose-coupled wheat germ agglutinin beads (Sigma catalogue number 61768), which were pre-equilibrated with wash buffer (0.1% Digitonin, 50 mM Tris-HCl, 500 mM NaCl, 64 mM Benzamidine, and 20 mM PMSF), and incubated for 2 hours at 4°C. Beads were pelleted, washed, and then eluted by incubating beads with Laemmli buffer in boiling water.

### Statistical analysis

All data are presented as mean ± standard error of the mean. Comparisons between groups was performed using a One-way ANOVA accompanied by a Tukey post hoc test to determine significance (p<0.05).

## Supporting Information

Figure S1 Localization of cytoplasmic actins in *mdx* quadriceps sections. (A) Ten micron thick sections from *mdx* quadriceps stained with laminin and cytoplasmic actin antibodies. Strong immunoreactivity was observed in what appeared to be macrophages invading necrotic fibers. (B) Ten micron thick sections from *mdx* quadriceps stained with a macrophage marker (Mac-1) and cytoplasmic actins. Colocalization between cytoplasmic actins and a macrophage marker (Mac-1) was observed.
(8.34 MB TIF)
