# Supplementary figures and images for: Skeletal Muscle-Specific Ablation of γcyto-Actin Does Not Exacerbate the mdx Phenotype
Source: PLoS One. 2008 Jun 11;3(6):e2419. doi: 10.1371/journal.pone.0002419 (PMC2409075; doi:10.1371/journal.pone.0002419)

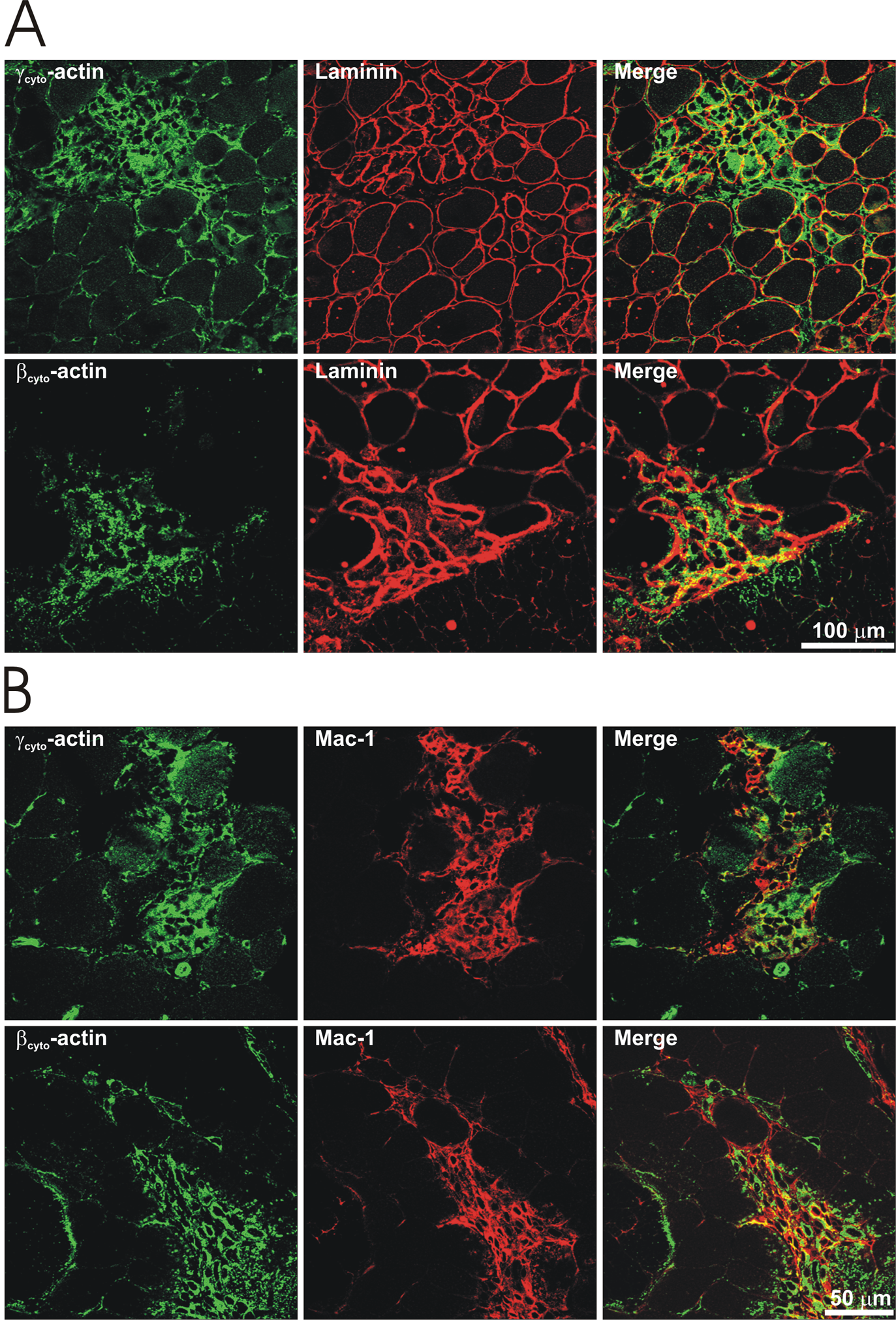

Supplement: Figure S1 — Localization of cytoplasmic actins in mdx quadriceps sections. (A) Ten micron thick sections from mdx quadriceps stained with laminin and cytoplasmic actin antibodies. Strong immunoreactivity was observed in what appeared to be macrophages invading necrotic fibers. (B) Ten micron thick sections from mdx quadriceps stained with a macrophage marker (Mac-1) and cytoplasmic actins. Colocalization between cytoplasmic actins and a macrophage marker (Mac-1) was observed. (8.34 MB TIF) [file pone.0002419.s001.tif]
